# 肺部小病灶经皮穿刺活检41例分析

**DOI:** 10.3779/j.issn.1009-3419.2018.09.06

**Published:** 2018-09-20

**Authors:** 牧 胡, 磊 刘, 坤 钱, 元博 李, 修益 支

**Affiliations:** 100053 北京，首都医科大学宣武医院胸外科 Department of Thoracic Surgery, Xuanwu Hospital, Capital Medical University, Beijing 100053, China

**Keywords:** 肺部, 小病灶, 活检, Lung, Small lesions, Biopsy

## Abstract

**背景与目的:**

近年来由于肺癌筛查工作的广泛开展，越来越多的肺部小病灶被发现，本研究旨在分析计算机断层扫描（computed tomography, CT）引导下经皮肺小病灶（直径 < 2 cm）穿刺活检结果及并发症、预后。

**方法:**

选择2015年1月-2017年12月期间行CT引导下经皮肺穿刺活检术41例肺内周围型病灶，单发病灶39例多发病灶2例，病灶最大径5-20（13.1±5.2）mm。病灶距离肺表面深度1-45（16.5±13.7）mm，毛玻璃样成分（ground glass opacity, GGO）比例0%-100%（66.8%±35.2%）。

**结果:**

41例患者进行43次穿刺。43次穿刺均成功取得病理组织。手术证实其中非典型腺瘤样增生（atypical adenomatous hyperplasia, AAH）3例，鳞癌1例，腺癌37例（原位癌7例，微侵润癌5例，侵润性25例，有2例患者为双原发癌），炎性病变2例。除2例炎性病变正在随访中，活检与手术病理吻合度（特异性）为100%。气胸22例，发生率51.2%，需闭式引流2例，17例发生咯血，发生率39.5%，均为自限性。2例出现颅内空气栓塞经治疗痊愈，发生率4.6%。

**结论:**

对于肺部小病灶CT引导经皮穿刺技术上可行。但对于肺部小病灶尤其是纯GGO的穿刺活检，目前还需要持谨慎态度。

## Abstract (en)

**Background and objective:**

When lung cancer screening work extensively developed in recent years, more and more small lung lesions were found in clinic. The aim of this study is to analysis computed tomography (CT) guided percutaneous biopsy for lung small lesions (diameter < 2 cm) on results, complications and prognosis.

**Methods:**

Choose CT guided percutaneous lung biopsy were performed in 41 cases of pulmonary peripheral lesions, single lesion in 39 cases, multiple lesions in 2 cases, 5-20 (13.1±5.2) mm in maximum diameter, depth from lung surface 1-45 (16.5±13.7) mm, ground-glass opacity (GGO) components 0%-100% (66.8%±35.2%).

**Results:**

41 patients and 43 biopsies successfully obtained pathological tissue. Atypical adenomatous hyperplasia in 3 cases, squamous carcinoma in 1 case, adenocarcinoma in 37 cases(carcinoma in situ in 7 cases, micro-invasive carcinoma in 5 cases, invasive adenocarcinoma in 25 cases, double primary lung cancer in 2 cases), inflammatory lesions in 2 cases. Except 2 cases of inflammatory lesions are in follow-up, biopsy and surgical pathology alignment (specificity) was 100%. 41 patients occurred complications related to percutaneous biopsy. Pneumothorax were in 22 cases, drainage required in 2 cases. There were 17 cases with hemoptysis, accounting for 39.5% incidence are self-limited. Intracranial air embolism occurred in 2 cases by 4.6% incidence. They were fully recovered.

**Conclusion:**

For small lung lesions, CT guided percutaneous biopsy is technically feasible. However, for small lung lesions especially pure GGO biopsy, it is still need to be cautious.

计算机断层扫描（computed tomography, CT）引导下经皮肺穿刺活检术是目前临床常用的获取肺部病变病理标本的方法，对临床治疗具有重要的指导意义。虽然该技术微创，但也存在着一定的风险，常见的并发症有气胸、出血和咯血。罕见并发症如体循环空气栓塞、针道种植转移、血胸等^[1-4]^。近年来由于肺癌筛查工作的广泛开展，越来越多的肺部小病灶被发现, 对于这些病灶的活检是难以回避的临床问题^[5, 6]^。本文就CT引导下肺小病灶（直径 < 2 cm）穿刺活检结果及并发症、预后予以分析，并提出对这一问题的思考。

## 1 资料与方法

### 1.1 一般资料

我院2015年1月-2017年12月期间行CT引导下经皮肺穿刺活检术的患者41例，其中男17例，女24例，年龄33-78（60.4±11.4）岁，肺内周围型病灶，单发病灶39例多发病灶2例，病灶最大径5-20（13.1±5.2）mm。病灶中右上肺9例、右下肺10例、右中叶4例、左上肺8例、左下肺12例。病灶距离肺表面深度1-45（16.5±13.7）mm，GGO成分比例0-100%（66.8%±35.2%）。患者均行痰找脱落细胞学检查提示未见肿瘤细胞，所有患者均无法从支气管镜检查获病理诊断。

### 1.2 术前准备

患者术前均常规行血常规、凝血功能、肝肾功、心电图等常规准备。明确无穿刺禁忌。术前与患者或者患者家属签穿刺知情同意书，告知患者手术过程并行呼吸屏气锻炼取得患者充分理解及配合。

### 1.3 穿刺方法

根据病灶位置和大小选择合适体位在预选出的穿刺点处固定上金属定位器。行CT扫描根据扫描结果确定最佳穿刺点、穿刺路径计算好进针角度、深度，于选定的穿刺点常规消毒、铺洞巾，2%利多卡因5 mL逐层麻醉至胸膜层，麻醉满意后应用穿刺活检针从选定穿刺点进针，按照既定角度、路径、深度进针至病灶再次行胸部CT平扫，确定穿刺针是否到达既定位置。假如不在既定位置可以根据CT扫描予以调整直至到达既定位置。拔出针芯接上活检枪，扳动扳机进行活检，留取穿刺组织标本用4%稀甲醛固定送病理学检查。必要时可以重复穿刺取组织。穿刺后平卧5 min，再次行胸部CT扫描观察有无气胸、肺内出血等并发症。穿刺病理为恶性患者限期手术治疗，穿刺病理为良性术后定期对患者进行随访。

## 2 结果

### 2.1 穿刺结果

41例患者进行43次穿刺。43次穿刺均成功取得病理组织。2例穿刺结果为炎性病变患者随访观察，其余39患者均接受胸腔镜手术切除。

### 2.2 病理学结果

明确病理学诊断43例。其中AAH 3例，鳞癌1例，腺癌37例（原位癌7例，微侵润癌5例，侵润性25例，有2例患者为双原发癌），炎性病变2例。除2例炎性病变正在随访中，活检与手术病理吻合度（特异性）为100%。

### 2.3 并发症

气胸22例，发生率51.2%，需闭式引流2例，引流术后痊愈。余20例少量气胸予以吸氧、卧床休息等处理后复查胸片完全吸收。17例发生咯血，发生率39.5%，均为自限性，予以止血治疗后痊愈。2例出现颅内空气栓塞，发生率4.6%，临床表现为一过性意识丧失、抽搐、失语、偏瘫，经抢救治疗后均痊愈。39例患者接受手术治疗，2例良性患者术后随访3个月未见其他并发症。

## 3 讨论

CT引导下的肺部病灶穿刺活检在临床工作中已经得到广泛开展^[7]^。随着活检装置的进步，目前临床采用的基本为自动或半自动组织切割活检针。细针抽吸活检基本已经较少采用。CT引导下经皮肺穿刺活检术诊断准确率89.39%，灵敏度85.11%，特异度100%^[8]^。随着穿刺经验的积累，对于小结节的穿刺在技术上已经成为可能。本中心的经验，在5 mm左右的结节技术上完全可以做到精确的取材。随着早期肺癌筛查的开展，越来越多的肺部小病灶被发现，这些小病灶的处理给临床工作带来了挑战^[5, 9, 10]^。穿刺活检和技术的进步和这一挑战恰逢其时。为此，笔者的中心对于小病灶穿刺进行了尝试并且得到初步结果。结果证实对于肺部小病灶穿刺获取满意的病理诊断在技术上已经成熟。穿刺的成功率100%，除2例良性结果需要随访进一步证实，活检病理与手术验证吻合度100%。

需要引起注意的一点是对于肺部小病灶穿刺活检的并发症问题。CT引导下经胸壁肺穿刺活检术的最常见的并发症是肺出血和气胸，其气胸发生率为10%-63%，肺出血发生率为26%-33%^[7, 11]^。这类并发症多属自限性，如本组穿刺后CT下可见气胸比例为51.2%，实际上需要闭式引流的仅2例，约5%。而针道和穿刺病灶的肺内出血在穿刺后CT表现上极为常见，但绝大多数均自限，无需特殊处理。

在本文的数据中空气栓塞的发生引起笔者极大的关注，43次穿刺出现2例空气栓塞，发生率为4.6%，虽然经过及时救治并痊愈，但过程相当危急。从文献报道情况来看。既往多数文献报道其发生率仅为0.02%-0.07%^[12-15]^，为罕见并发症。然而，随着对其认识的增多，部分研究者提出其发生率可高达0.21%-0.4%^[16-18]^，只是其中很大一部分无症状患者未被发现。即便如此，本研究4.6%的空气栓塞发生率远高于文献数据。细细探究原因，笔者认为很可能是由于穿刺对象的不同的结果。文献报道中穿刺的病灶大小大部分多在20 mm以上且实性居多，而本文的穿刺病灶均在20 mm以下，有部分病灶仅仅只有5 mm左右，且大部分病灶为部分实性，部分为纯GGO。肺穿刺活检并发的体循环空气栓塞气体进入肺静脉的可能途径有：①空气经穿刺针直接进入肺静脉：穿刺套管针刺破肺静脉后拔出针芯，套管暴露于空气中，形成空气-肺静脉直接相通。②肺内空气经穿刺道进入肺静脉：GGO多有较丰富的血管且含有肺泡，当穿刺针穿过GGO中肺泡组织且同时刺伤邻近肺静脉时，即可形成气道-肺静脉瘘。若患者出现深吸气、咳嗽时，空气即可经穿刺针道进入肺静脉。其中，肺内空气经穿刺道进入肺静脉是目前文献所报道的绝大多数病例的可能发病机制^[19]^。因此，病灶的性质应该是空气栓塞的高危因素之一。肺穿刺合并空气栓塞积极治疗后部分患者可以痊愈，但仍有大部分患者预后较差。Ibukuro等^[17]^研究的19例患者中5例死亡，死亡率达26.3%。随着认识的提高和高压氧的广泛应用，近年来死亡率呈明显下降趋势，但有文献报道非致命空气栓塞患者合并长期神经系统功能障碍者高达50%^[12]^。

从文献检索情况来看，近几年来空气栓塞的报道呈现明显上升趋势，这和肺癌筛查的关系耐人寻味。虽然从技术上肺部小病灶的穿刺已经可以做到，但是并发症，尤其是空气栓塞这一严重并发症的危险需要充分考虑。近年来，胸外科、呼吸科和影像介入科都在对肺部小病灶的新课题进行相关的研究，活检、手术、立体定向放疗、射频消融包括密切观察均是对高危病灶的可选手段。对于肺部小病灶尤其是纯GGO的穿刺活检，目前还需要持谨慎态度。
